# Normative values for calf muscle strength-endurance in the general population assessed with the Calf Raise Application: A large international cross-sectional study

**DOI:** 10.1016/j.bjpt.2025.101188

**Published:** 2025-02-27

**Authors:** Tjerk SO Sleeswijk Visser, Seth O’ Neill, Kim Hébert-Losier, Denise Eygendaal, Robert-Jan de Vos

**Affiliations:** a Department of Orthopedics and Sports Medicine, Erasmus MC University Medical Centre, Rotterdam, , The Netherlands; b School of Healthcare, Department of Life Sciences, University of Leicester, United Kingdom; c Te Huataki Waiora School of Health, The University of Waikato, Adams Centre for High Performance, Mount Maunganui, Tauranga, New Zealand

**Keywords:** Achilles tendon, Heel rise, HRET, Reference

## Abstract

**Background:**

Calf muscle strength-endurance can be reliably assessed with the Heel Rise Endurance Test (HRET), but normative values are lacking.

**Objectives:**

To establish normative values for calf muscle strength-endurance, adjusted for personal characteristics.

**Methods:**

500 individuals without current/previous symptoms of Achilles tendinopathy or recent lower limb immobilization were included. Primary outcome measures were the number of repetitions, total work (J), total vertical displacement (cm), and peak height (cm) upon the single-leg HRET, assessed using the validated Calf Raise Application. A multiple quantile regression model was developed, incorporating covariates (personal characteristics) which significantly impacted HRET metrics. Median (50.0th percentile) and 95 % reference intervals (2.5th-97.5th percentiles) were derived.

**Results:**

55 % of the participants were female and 88 % participated in physical activities. Median (dominant/non-dominant leg) number of repetitions was 25/24, total work was 1374/1325 J, vertical displacement was 192/186 cm, and peak height was 9.3/9.6 cm. There was no significant difference between the dominant and non-dominant leg for any HRET metric. Lower physical activity levels, female sex, and higher body mass index (BMI) were associated with lower HRET metrics.

**Conclusions:**

Normative calf muscle strength-endurance metrics (number of repetitions, total work, total displacement, and peak height) were developed. Personal characteristics influence HRET outcomes, with female sex, higher BMI, and lower physical activity levels being associated with lower HRET metrics. An openly accessible calculator for estimating normative HRET metrics was developed to help healthcare providers monitor personalized recovery trajectories and provide well-informed rehabilitation guidance. Documenting HRET metrics beyond repetition count may aid in assessing impairment severity and evaluating calf muscle function.

## Highlights

• Outcomes of the HRET are influenced by personal characteristics.
• Female sex, higher BMI, and low activity level link to lower HRET metrics.
• Normative values may help track recovery and guide rehabilitation plans.
• Online tool for HRET estimates available at: www.achillestendontool.com/HRET.

## Introduction

The strength-endurance of the plantar flexors is frequently assessed in clinical practice and research using the single-leg heel rise endurance test (HRET).^1^^,^^2^ The results obtained from this test are valuable for evaluating impairment severity, tracking recovery of Achilles tendon injuries, assessing exercise program effects on functional abilities, and guiding return-to-sport recommendations.^2^, ^3^, ^4^

Normative values of tests are often used as a reference for evidence-based clinical practice.^5^ The contralateral limb cannot always be used as a reference for comparison as it does not always reflect optimal function.^6^^,^^7^ Consequently, it is important to have HRET normative values for both limbs. The existing literature suggests that a "normal" HRET performance comprises approximately 20 heel raises for children^8^ and 25 heel raises for adults, with age, sex, body mass index (BMI), and activity level influencing the results in Swedish individuals.^6^^,^^9^ Although the HRET has good test-retest reliability, a limitation of the test is that it relies on the total number of repetitions performed, without taking into account the quality of movement.^6^^,^^10^ For example, individuals can complete numerous repetitions, but not raise the heel very high. More objective metrics, such as total work or peak height, are considered scientifically more robust than the number of repetitions and are deemed important measures of calf muscle tendon unit function.^11^, ^12^, ^13^, ^14^ The recently developed Calf Raise Application can reliably assess these metrics.^15^ However, normative values for these HRET metrics in the general population are lacking.^2^^,^^13^

The primary objective of this study was to establish normative HRET values in a large population of healthy individuals, using objective metrics such as number of repetitions, total work (J), total displacement (cm), and peak height (cm). The secondary aim was to assess how HRET metrics are influenced by personal characteristics, including age, sex, BMI, and activity level.

## Methods

### Study design

The study was designed at the Erasmus MC University Medical Centre (NL) in collaboration with the University of Leicester (UK) and the University of Waikato (NZ). The local Medical Ethics Committee (Southwest-Holland, The Netherlands) approved the study protocol (MEC-2020–0585). The trial was prospectively registered (Netherlands Trial Register, NL9010) and we adhered to the Strengthening the Reporting of Observational Studies in Epidemiology (STROBE) guidelines.^16^

### Protocol deviations

In the initial study registration, 'total work' and 'total vertical displacement' were listed as secondary outcomes, but were reclassified as primary outcomes. Similarly, 'repetitions' and 'peak height,' were not initially registered, but were included as primary outcome measures. These decisions were based upon further clinical assessment and post-registration discussions (during the data-collection phase) within our research team. The correlation between structure and calf muscle strength-endurance was prospectively registered but can't be reported at this stage as it requires specific software, which, as of now, has not been developed and validated by the manufacturer.

### Participants

The study was conducted at the outpatient departments of two large universities (Erasmus MC University Medical Centre and University of Leicester) from October 2020 to June 2023. The study was paused from November 2020 to May 2022 due to Covid-19 restrictions. These restrictions also compelled us to limit sample size to 500, which is a reduction of 100 participants compared to the pre-defined protocol.

We aimed to include a sample of participants that accurately reflects the general population, with an even distribution of both sex and across decades of life. To recruit participants, a comprehensive announcement was disseminated through internal websites and various social media platforms, including Twitter (now X), Facebook, and LinkedIn. Interested individuals were screened remotely. Those meeting eligibility during remote screening were scheduled for an appointment with a researcher, during which further screening assessments were conducted.

Inclusion criteria were: (1) at least 18 years of age, (2) no current or prior history of Achilles tendon pain or stiffness, (3) no localized fusiform thickening of the Achilles tendon on palpation, and (4) a full score on the adapted (questions 1 to 5) Victorian Institute of Sports Assessment-Achilles (VISA-A) questionnaire.^17^^,^^18^ Exclusion criteria were: (1) a history of Achilles tendon or ankle surgery, (2) any lower-limb injury requiring immobilization within the past 12 months, and (3) known systemic inflammatory disorders/internal diseases that may cause Achilles tendon abnormalities.

Eligible participants were asked to sign an informed consent form before data collection. Subsequently, participants were asked to fill out a more comprehensive survey to collect demographic data (age, sex, height, mass, and BMI), health status details (presence of comorbidities, smoking, medication use) and sports activities information. Physical Activity Level (PAL) was assessed using a 6-point Likert scale.^6^ Leg dominance was assessed by asking participants the leg that would be used to kick a ball or the leg with which a participant would step forward when gently pushed from behind.^19^

### Procedures

**Fig. 1:**
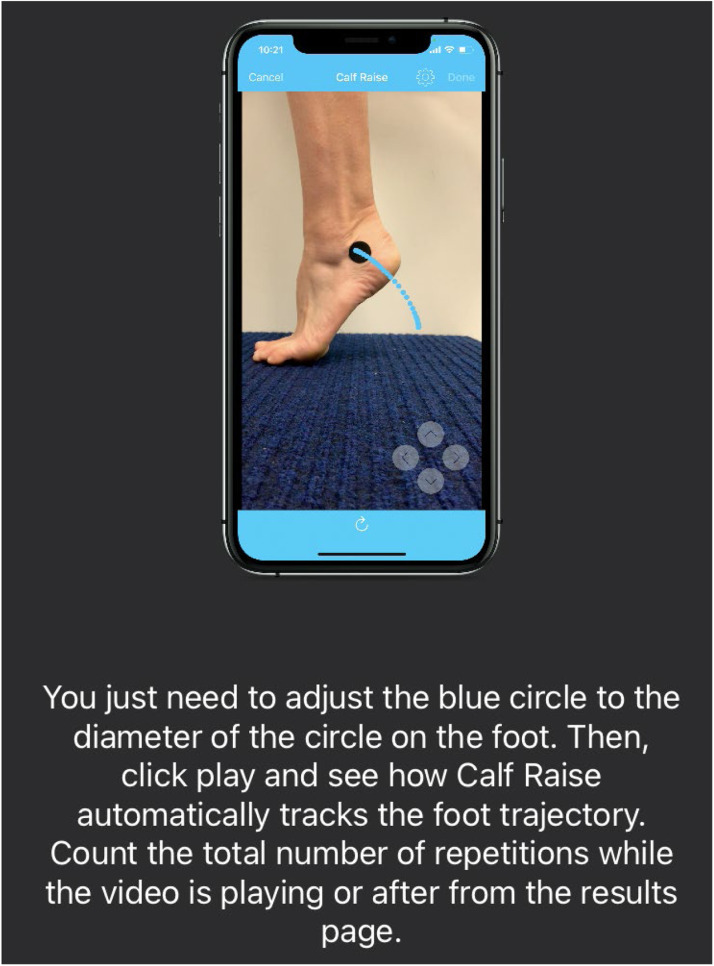
Example of the Calf Raise Application. A round sticker with a diameter of 2.5 cm is placed just below the distal tip of the lateral malleolus. The Calf Raise Application tracks this sticker using computer vision algorithms. As the participant goes up, the heel with the adjusted sticker moves upwards and forwards in the screen.

After completing the survey, participants performed the HRET. During this test, participants were instructed to assume a single-leg stance on a 10° incline board barefoot with the knee in full extension and the trunk erect. Video recordings of each HRET were obtained using the specialized Calf Raise Application.^15^^,^^20^ A device (IPad or IPhone, Apple Inc., Cupertino, United States) was used to record the test (Fig. 1). The device was placed upright in a fixed position (in a stand on a flat base) to the side of participants and at a standardized distance of 30 cm away from the participants’ foot in a way that allowed the entire foot to be visible during the HRET.^15^ For longer feet the recording device was moved up to 50 cm away from the participants’ foot.^21^

Participants were allowed fingertip support at shoulder height on the wall in front of them. The objective of the test was to raise the heel as high as possible on each repetition, returning it to the incline board, and performing as many repetitions as possible while minimizing anterior movement. A digital metronome set at 60 beats per minute guided the test, with participants ascending on one beat and descending on the next (i.e., 30 repetitions per minute). To acclimate to the metronome pace, each participant performed 10 bilateral standing heel rises as a warm-up prior to testing.

Participants were informed of the test termination criteria: 1) the heel can no longer be lifted from the incline board, 2) the pace of the digital metronome can no longer be followed, 3) the knee angle or trunk position can no longer be maintained, or 4) more than fingertip support on the wall is needed for balance. If a termination criterion was observed, a verbal prompt was given. Testing was terminated when there was no response to two consecutive prompts. Throughout the testing procedure, participants received verbal instructions to maintain the specific parameters, including heel excursion, cadence, balance support, and knee angle. The HRET was conducted once on each leg, and the order of testing was quasi-randomized, based on the moment of inclusion (participant #1 started on the right leg, participant #2 on the left leg, etc.). A 2-minute rest period was provided to participants following the completion of the first single-leg HRET, after which the test was repeated using the opposite limb.

### Outcome measures

By tracking the vertical displacement of the round sticker on the foot of participants and based on the mass (kg) of individuals, the Calf Raise Application calculates various metrics. The primary outcome measures in this study were the number of repetitions, total positive work (J), total vertical displacement (cm), and peak height (cm). These Calf Raise Application metrics are validated and show excellent reliability.^15^^,^^21^ Secondary outcome measures, as reported in the application, were vertical height loss (%) and peak power (W). The exact working mechanism and validation of the Calf Raise Application have been described in detail elsewhere.^15^^,^^21^

### Statistical analysis

Given the objective of establishing normative values and the skewed distribution of the data (as assessed with the Kolmogorov-Smirnov Test), we used quantile regression for the analysis as it estimates medians without imposing distributional assumptions. Potential differences in HRET metrics between the dominant and non-dominant leg were analysed using a Mann-Whitney U test. When no statistically significant differences were observed, mean HRET metrics were used to develop the quantile regression model. Initially, bivariate models were constructed (using Spearman's correlation coefficient) to examine the relationship between each covariate (age, sex, height, mass, BMI, leg dominance, and PAL) and the HRET metrics. Subsequently, a multiple quantile regression model was developed, incorporating the covariates that demonstrated a significant influence on HRET metrics. The median (50.0th percentile), lower (2.5th), and upper (97.5th) percentile values of the regression models were extracted to present HRET metrics as median with a 95 % (2.5th to 97.5th percentiles) reference interval (RI). A covariate was excluded from the multiple regression model if the following two criteria were met: 1) it exhibited a weak/negligible (*r* < 0.3)^22^ correlation coefficient and 2) the removal of the covariate did not impact the model's 'accuracy,' as indicated by the stability of the R^2^ value. To evaluate the influence of each covariate on HRET metrics, the 95 % confidence intervals (CI) were extracted to allow estimation of the impact of each covariate. We adhered to the CHecklist for statistical Assessment of Medical Papers (CHAMP) statement for the statistical analysis and presentation of results.^23^ IBM SPSS Statistics (version 28.0.1.0) were used for all analyses, and significance was set at *p* < 0.05.

## Results

### Flow of participants through the study

A total of 547 asymptomatic persons were screened for eligibility and 500 of these participants were included (*n* = 300 at Erasmus MC and *n* = 200 at the University of Leicester). A flowchart of the study is presented in Supplementary material online (Supplementary Figure 1). In five participants, the HRET metrics could not be extracted due to recording/technical errors; there was a complete dataset for 495 participants.

The participant characteristics are presented in Table 1. More than half of the participants were female (55 %) and the majority (88 %) participated in sports activities. Eight participants did not want to disclose their sex. The age of the participants ranged between 18 and 81 years.

**Table 1:** Participant characteristics. Values are medians [IQR] unless otherwise described.

Participant characteristics (*n* = 500)	Median [IQR]		
	Overall	Female	Male
**Population demographics**			
Age (years)	30 [22 - 50]	34 [23 - 52]	28 [21 - 45]
Sex (Male/Female/Other; n)	218/274/8	274	218
Height (cm)	174 [168 - 180]	169 [164 - 173]	181 [176 −186]
Mass (kg)	71 [64 - 80]	66 [60 - 73]	78 [72 - 87]
BMI (kg/m^2^)	23.3 [21.7 - 25.6]	23.1 [21.3 - 25.4]	23.6 [22.2 - 26.0]
Leg dominance (Right/Left/Both/unknown)	442/46/10/2	234/30/8/2	198/18/2/0
**General health and comorbidities**			
Sports participation (yes/no)	439/61	234/40	197/21
Physical Activity Level (PAL; 1–6)	5 [4 - 6]	5 [4 - 5]	5 [4 - 6]
Medication use (yes/no; n)	128/372	86/188	41/177
Smoking (never/current/stopped)	439/28/33	245/13/16	187/15/16
Alcohol consumption (units/week)	4 [1 - 8]	3 [1 - 6]	5 [2 - 10]
Comorbidities* (yes/no; n)	52/448	37/237	15/203

Abbreviations: **BMI**, Body Mass Index (kg/m^2^); **PAL**, Physical Activity Likert Scale; 1–6, 1 = Hardly any physical activity, 2 = Mostly sitting, sometimes walk, easy tasks/play, 3 = Light physical activity for about 2–4 times a week (e.g., fishing, talking, dancing), 4 = Moderate exercise 1–2 h a week (jogging, swimming, gymnastics), 5 = Moderate exercise at least 3 h a week (jogging, swimming, gymnastics), 6 = Hard or very hard exercise regularly and several times a week during which the physical exercise is great (jogging, rugby, football).

⁎ Comorbidities included: diabetes, hypercholesterolemia, hypertension, heart/vessel diseases, and thyroid disease.

### Normative values for HRET metrics

The median (95 % RI) number of repetitions completed was 25 (13–50) for the dominant leg and 24 (12–51) for the non-dominant leg. The normative values (median, 95 % RI) for the number of repetitions, total work, total displacement, and peak height are presented in Table 2. The normative values for the secondary outcome measures are presented in Supplementary material online (Supplementary Table 1) as well as the normative data for the right and the left leg (Supplementary Table 2).

**Table 2:** Normative data for the HRET metrics of the primary outcome measures. Data in this table are presented for 483 participants as 10 participants reported no leg dominance, leg dominance was unknown in 2 participants and HRET metrics could not be extracted due to technical errors in 5 participants.

Normative values HRET metrics (*n* = 483)	Median (95 % RI)*		
	Overall	Female	Male
**Dominant leg**			
Repetitions (n)	25 (13 – 50)	24 (13 – 43)	26 (14 – 53)
Total work (Joule)	1374 (609 – 2676)	1204 (608 – 2151)	1676 (623 – 2935)
Total displacement (cm)	192 (86 – 376)	182 (84 – 343)	210 (91 – 405)
Peak height (cm)	9.3 (5.2 – 13.0)	9.2 (5.5 – 12.5)	9.4 (4.9 – 13.9)
**Non-dominant leg**			
Repetitions (n)	24 (12 – 51)	23 (11 – 48)	26 (13–62)
Total work (J)	1325 (539 – 2786)	1130 (488 – 2098)	1623 (662 – 3168)
Total displacement (cm)	186 (84 – 380)	174 (75 – 361)	204 (100 – 448)
Peak height (cm)	9.6 (5.6 – 13.8)	9.4 (5.4 – 12.7)	10.1 (5.7 – 14.1)

Abbreviations: HRET, Heel Rise Endurance Test; RI, Reference Interval.

⁎ Values are median with 95 % reference interval (2.5th and 97.5th percentile).

There was no statistically significant difference between the dominant and the non-dominant side for any of the HRET metrics nor a correlation between leg dominance and HRET metrics (Supplementary Tables 3 and 4). Bivariate analyses revealed that there was a significant correlation between sex (*r* = 0.20, *p* < 0.001), height (*r* = 0.17, *p* < 0.001), BMI (*r*=−0.28, *p* < 0.001), and PAL (*r* = 0.23, *p* < 0.001) and the number of repetitions. Total work significantly correlated with sex (*r* = 0.45, *p* < 0.001), height (*r* = 0.52, *p* < 0.001), mass (*r* = 0.33, *p* < 0.001), and PAL (*r* = 0.19, *p* < 0.001). Sex (*r* = 0.23, *p* < 0.001), height (*r* = 0.26, *p* < 0.001), BMI (*r*=−0.32, *p* < 0.001), and PAL (*r* = 0.21, *p* < 0.001) significantly correlated with total displacement. There was a significant correlation between sex (*r* = 0.12, *p* = 0.009), height (*r* = 0.20, *p* < 0.001), and BMI (*r*=−0.15, *p* < 0.001) with peak height. Results of the bivariate analyses for the secondary outcome measures are presented in Supplementary material online. The results of the multiple quantile regression model with estimates of the relevant parameters on the different HRET metrics are displayed in Table 3 (and Supplementary Table 5 for the secondary outcome measures).

**Table 3:** Estimates (95 % CI) of the effect of the parameters on the different HRET metrics derived from the multiple quantile regression analysis adjusted for age, height (cm), mass, BMI, sex, and physical activity level (PAL). Examples on how to employ the normative equations based on two fictional patients are provided in the lower part of the table.

Parameter	Repetitions	Total work	Vertical displacement	Peak height
Intercept	41.2 (35.4, 47.0)	−2084 (−3329, −839)	156.7 (−25.7, 339.2)	3.3 (−2.4, 9.0)
Age	XX	XX	XX	XX
Height	††	19.5 (12.1, 27.0)	1.0 (0.8, 2.0)	0.044 (0.015, 0.074)
BMI	−0.61 (−0.84, −0.38)	XX	−5.5 (−7.4, −3.5)	−0.069 (−0.13, −0.10)
Mass	XX	2.6 (−2.0, 7.3)	XX	XX
Sex	−2.7 (−4.4, −1.1)	−164.3 (−296.8, −31.8)	−12.8 (−31.2, 5.7)	††
PAL 2	3.6 (−3.8, 10.9)	−211.4 (−649.9, 227.1)	−18.7 (−79.8, 42.4)	XX
PAL 3	−2.6 (−5.4, 0.27)	−185.3 (−354.1, −16.6)	−29.4 (−52.9, −5.9)	XX
PAL 4	−0.93 (−3.3, 1.4)	−135.6 (−275.8, 4.6)	−22.4 (−41.9, −2.8)	XX
PAL 5	0.43 (−1.6, 2.5)	33.9 (−86.2, 154.1)	5.9 (−10.9, 22.6)	XX

Normative equation*	Intercept + height + BMI/mass + sex + PAL			
Example A	Female, 53 years, 29 kg/m^2^, 165 cm, 79 kg, PAL 3			
Example B	Male, 25 years, 21 kg/m^2^, 184 cm, 71 kg, PAL 5			
**Repetitions**⁎⁎	41.2 – 0.61 x (BMI) – 2.7 x (sex) + PAL			
A: 18 (12 – 33)	41.2 – 0.61 x (29) −2.7 x (1) – 2.6			
B: 29 (16 – 55)	41.2 – 0.61 x (21) −2.7 x (0) + 0.43			
**Total work**⁎⁎	−2084 + 19.5 x (height) + 2.6 x (mass) – 164.3 x (sex) + PAL			
A: 989.3 (531.7 - 1651.0) J	−2084 + 19.5 x (165) + 2.6 x (79) – 164.3 x (1) – 185.3			
B: 1732.5 (824.6 - 3108.2) J	−2084 + 19.5 x (184) + 2.6 x (71) – 164.3 x (0) + 33.9			
**Vertical displacement**⁎⁎	156.7 + 1.0 x (height) – 5.5 x (BMI) −12.8 x (sex) + PAL			
A: 126 (69 - 246) cm	156.7 + 1.0 x (165) – 5.5 x (29) – 6.5 x (1) – 29.4			
B: 237 (117 - 430) cm	156.7 + 1.0 x (184) – 5.5 x (21) – 6.5 x (0) + 5.9			
**Peak height**⁎⁎	3.3 + 0.044 x (height) – 0.069 x (BMI)			
A: 8.6 (5.0 - 11.7) cm	3.3 + 0.044 x (165) – 0.069 x (29)			
B: 9.9 (5.8 - 13.6) cm	3.3 + 0.044 x (184) – 0.069 x (21)			

Abbreviations: **BMI,** Body Mass Index; **PAL,** Physical Activity Level.

⁎ Sex: male = 0, female = 1, PAL 6 = 0.

⁎⁎ Values are median (mm) with 95 % RI (2.5th percentile, 97.5th percentile).

†† Covariate removed from the multiple quantile regression model as it exhibited a weak/negligible (*r* < 0.3)^22^ correlation coefficient and the removal of the covariate did not impact the model's 'accuracy,' as indicated by the stability of the R^2^ value.

## Discussion

In this large international cross-sectional study, we presented normative values for HRET metrics in healthy individuals with a large age range, adjusted for personal characteristics. We found that the median number of repetitions and peak height was 25 and 9.3 cm for the dominant leg and 24 and 9.6 cm for the non-dominant leg. There was no significant difference between the dominant and the non-dominant leg for any of the HRET metrics. Lower physical activity levels, female sex, lower body height, and higher BMI were associated with lower HRET metrics. For the primary outcome measures, we found no correlation between age and HRET metrics.

This study presents novel normative values for HRET metrics. The median number of repetitions achieved in the present investigation corresponds with previous findings.^6^^,^^21^^,^^24^ Various studies have reported mean values for total work (ranging from 1800 to 3000 J) or peak height (ranging from 9 to 14.1 cm) in the uninjured legs of patients recovering from Achilles tendon rupture^12^^,^^24^^,^^25^ or a small (38 participants) sample of healthy individuals.^11^ Our median values for work (1380 J) and peak height (9.7 cm) are at the lower end of this spectrum. This discrepancy can potentially be attributed to the relatively small (38 – 96 participants) or selected (very active) study populations that are younger in age in previously published studies. The primary factor contributing to the observed variance in results is likely the methodology employed in the current study for collecting calf raise data, specifically the use of a marker placed below the lateral malleolus^15^ rather than on the heel, as done in the aforementioned studies to attach a linear encoder.^11^^,^^12^^,^^24^ This below malleolus placement is found to be more valid when using the Calf Raise Application,^15^ but results in relatively lower values compared to using a marker positioned on the heel.^15^^,^^26^ It is important to note that the current and aforementioned studies used a 10° incline board which is common in research but may not always be available in clinical practice. This should be considered when interpreting results of the HRET performed on a flat surface compared to the finding of the current study as the use of a flat surface may result in lower HRET outcomes due to less vertical height displacement.

Our results show that HRET metrics are influenced by personal characteristics. The findings that lower physical activity, higher BMI, lower body height, and female sex are associated with lower HRET outcomes are consistent with previous findings.^6^ We did not observe a correlation between age and the number of repetitions, which contrasts to earlier work showing a significant decline in number of repetitions for each passing decade of life.^6^ A possible explanation for this may be that, despite the efforts to include a balanced population with regards to age and sex, the study population was relatively young with a mean (min-max) age of 36 (18–81) years as well as relatively physically active (Supplementary Figures 2 and 3). This relatively young and active population limit the generalizability of our findings. It is likely that other personal factors influence the results, like motivation^27^ and self-confidence. We did encourage participants to perform maximally, but we are aware that psychological factors – which we did not consider – may affect outcome.

### Clinical implications

Literature shows inconsistent findings with regards to the influence of leg dominance on the number of repetitions. While some studies reported no between-leg differences,^6^^,^^28^ others reported the non-dominant side to exhibit greater strength^29^ or a higher number of repetitions than the dominant side.^30^ This observation could potentially be attributed to different definitions of leg dominance being used.^19^^,^^30^ The current study did not find any difference between leg dominance and HRET performance. The inconsistent evidence makes it difficult to support or refute the use of the uninvolved side as a reference for comparison when evaluating HRET performance in clinical practice. This issue becomes particularly apparent in injured individuals where is it known that HRET performance is negatively impacted in both limbs.^6^ To address this issue, clinicians may benefit from knowledge of normative values, adjusted for personal characteristics. We have developed an openly accessible web-based calculator for estimating normative HRET metrics (www.achillestendontool.com/HRET). This tool may be valuable for clinicians to monitor personalized trajectories of recovery and to provide well-informed rehabilitation guidance.

### Strengths and limitations

The strengths of this study lay in the design, with the inclusion of a large and international study population, a pre-defined protocol, the use of a validated openly accessible application to obtain outcome measures and the development of the open access tool for calculating normative HRET values, adjusted for personal characteristics to facilitate implementation in clinical practice. There are several limitations to this study. First, while the age range of the included participants was broad, the mean age of the study population was relatively young. This age may have led to the underestimation or absence of a correlation between age and HRET performance. Second, the normative values were derived exclusively from the Calf Raise Application. This application has demonstrated excellent validity and reliability, but our findings may not translate directly to normative values for other methods of assessing the HRET (such as the use of a linear encoder placed on the heel) or testing in different positions (e.g., no 10° incline or with shoes on) or with different cadence. However, this application is free for use and easily accessible for clinical and research use. Thirdly, during the design phase of the study we decided to collect the peak power and vertical height loss as outcome measures. Peak power is less relevant, because we used a metronome, resulting in more constant peak power values. Vertical height loss is known to be the least reliable and valid outcome measure.^15^ We therefore present these metrics as secondary outcome measures.

## Conclusion

Normative calf muscle strength-endurance metrics (number of repetitions, total work, total displacement, and peak height were developed. Outcomes of the single-leg HRET are influenced by personal characteristics, with female sex, higher BMI, lower body height, and lower physical activity levels being associated with lower HRET metrics. We have established normative values for various HRET metrics (www.achillestendontool.com/HRET). Documenting other important measures of calf muscle tendon unit function beyond repetition count may help clinicians in determining the severity of impairment or to evaluate treatment outcomes.

## Declaration of competing interest

One of the authors (KHL) is the developer of the free-to-use Calf Raise Application. The data from the application were obtained from independent researchers not related to the development of the application.

## Appendix Supplementary materials

Image, application 1
