# Supplementary material for: Normative values for calf muscle strength-endurance in the general population assessed with the Calf Raise Application: A large international cross-sectional study
Source: Braz J Phys Ther. 2025 Feb 27;29(3):101188. doi: 10.1016/j.bjpt.2025.101188 (PMC11923623; doi:10.1016/j.bjpt.2025.101188)
Supplement: Supplementary file 1 [file mmc1.pdf]

SUPPLEMENTARY FILE 1.

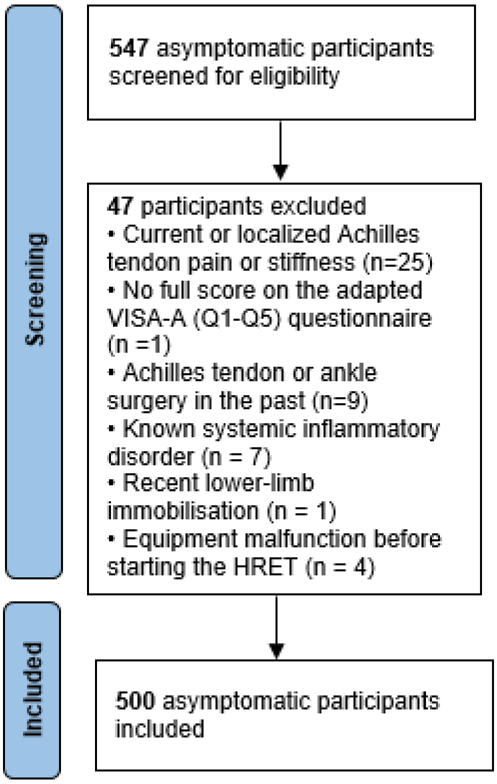

Supplementary Figure 1. Flowchart of the study.

Supplementary Table 1. Normative values (median, 95% reference interval) of the secondary outcome measures (vertical height loss (%) and peak power (Watt)). Data in this table are presented for 483 participants as 10 participants reported no leg dominance, leg dominance was unknown in 2 participants, and HRET metrics could not be extracted due to technical errors in 5 participants.

Normative values HRET metrics (n=483)	Median (95% RI)*		
	**Overall**	**Female**	**Male**
**Dominant**			
Peak power (Watt)	242 (132, 463)	217 (120, 377)	286 (152, 521)
Vertical height loss (%)	22.1 (0.7, 73.2)	20.2 (0.5, 64.3)	24.0 (1.0, 77.4)
**Non-dominant**			
Peak power (Watt)	249 (124, 476)	220 (118, 445)	284 (169, 522)
Vertical height loss (%)	20.5 (0.9, 71.6)	19.2 (0.8, 74.9)	20.9 (1.3, 70.3)

Abbreviations: HRET, Heel Rise Endurance Test; RI, Reference Interval

* Values are median with 95% reference interval (2.5th and 97.5th percentile)

Supplementary Table 2. Normative data for the HRET metrics for the left leg and right leg.

Normative values HRET metrics (n=495)	Median (95% RI)*		
	**Overall**	**Female**	**Male**
**Left**			
Repetitions (n)	24 (11, 51)	23 (11 , 46)	26 (13, 59)
Total work (J)	1304 (537, 2743)	1125 (467, 2097)	1622 (663, 3024)
Total displacement (cm)	185 (80, 379)	172 (74, 353)	206 (97, 418)
Peak height (cm)	9.7 (5.5, 13.8)	9.4 (5.4, 12.8)	10.1 (5.5, 14.0)
Peak power (Watt)	249 (124, 508)	213 (118, 436)	281 (169, 541)
Vertical height loss (%)	20.7 (1.0, 71.5)	19.4 (0.9, 73.1)	20.9 (1.3, 70.3)
**Right**			
Repetitions (n)	25 (13, 52)	24 (13, 46)	27 (14, 55)
Total work (Joule)	1379 (599, 2755)	1203 (544, 2147)	1676 (623, 3094)
Total displacement (cm)	191 (86, 377)	178 (83, 347)	209 (91, 431)
Peak height (cm)	9.3 (5.2, 13.0)	9.2 (5.5, 12.5)	9.4 (4.9, 14.0)
Peak power (Watt)	242 (136, 447)	218 (131, 387)	290 (152, 502)
Vertical height loss (%)	21.4 (0.7, 72.4)	19 (0.5, 65.9)	23.9 (1.0, 77.4)

Abbreviations: HRET, Heel Rise Endurance Test; RI, Reference Interval. * Values are median with 95% reference interval (2.5th and 97.5th percentile)

Supplementary Table 3. Difference in HRET metrics between the dominant and the non-dominant leg for both sides.

**N = 483**	**Mean difference**	**P-value**
**Left (n=46)**		
Repetitions (n)	-1.5	0.517
Total work (Joule)	-93.2	0.211
Total displacement (cm)	-17.9	0.164
Peak height (cm)	-0.05	0.886
Peak power (Watt)	-0.4	0.905
Vertical height loss (%)	-0.16	0.398
**Right (n=437)**		
Repetitions (n)	-0.3	0.641
Total work (Joule)	-8.5	0.608
Total displacement (cm)	-6.1	0.311
Peak height (cm)	0.02	0.944
Peak power (Watt)	14.6	0.205
Vertical height loss (%)	-1.6	0.567

Abbreviations: HRET, Heel Rise Endurance Test

Supplementary Table 4. Correlation between leg dominance and the Heel Rise Endurance Test (HRET) metrics for the right and left leg separately.

**N = 483**	Correlation coefficient	p-value
**Left (n=46)**		
Number of repetitions	-0.010	0.820
Peak height	0.003	0.941
Total displacement	0.020	0.655
Total work	0.013	0.776
Peak power	-0.033	0.472
Vertical height loss	-0.074	0.104
**Right (n=437)**		
Number of repetitions	-0.008	0.863
Peak height	-0.024	0.592
Total displacement	0.005	0.917
Total work	-0.017	0.715
Peak power	-0.102	0.025
Vertical height loss	-0.012	0.792

Bivariate analyses for the secondary outcome measures

There was no significant correlation between any of the HRET metrics and vertical height loss. Age (r=-0.12, p=0.010) sex (r=0.49, p<0.001), height (r=0.52, p<0.001), weight (r=0.55, p<0.001), and physical activity level (r=0.12, p=0.009) significantly correlated with peak power.

Supplementary Table 5. Estimates (95% CI, p-value) of the effect of the parameters on the different Heel Rise Endurance Test (HRET) metrics for the secondary outcome measures, derived from the multiple quantile regression analysis adjusted for age, height (cm), mass, BMI, sex, and physical activity level (PAL). An example on how to employ the normative equations based on two fictional patients is provided in the lower part of the table.

Parameter	Peak power
Intercept	-175.5 (-337.9, -13.1)
Age	-0.69 (-1.1, -0.28)
Height	1.7 (0.69, 2.6)
BMI	XX
Mass	2.4 (1.8, 2.9)
Sex	-17.8 (-35.0, -0.5)
PAL 2	-64.6 (-121.7, -7.6)
PAL 3	-2.5 (-25.1, 20.1)
PAL 4	-2.5 (-21.2, 16.2)
PAL 5	-4.8 (-20.8, 11.2)

Normative equation*	Intercept + age + height + mass + sex + PAL
Example A	Female, 53 years, 29 kg/m^2, 165 cm, 79 kg, PAL 3
Example B	Male, 25 years, 21 kg/m^2, 184 cm, 71 kg, PAL 5
**Peak Power****	-175.5 – 0.69 x (age) + 1.7 x (height) + 2.4 x (mass) – 17.8 x (sex) + PAL
A: 232.1 (146.1 - 302.9) W	-175.5 – 0.69 x (53) + 1.7 x (165) + 2.4 x (79) – 17.8 x (1) – 2.5
B: 285.7 (175.0 - 485.3) W	-175.5 – 0.69 x (25) + 1.7 x (184) + 2.4 x (71) – 17.8 x (0) – 4.8

* Sex: male = 0, female = 1, PAL 6= 0

** Values are median (mm) with 95% RI (2.5th percentile, 97.5th percentile)

Abbreviations: **BMI,** Body Mass Index; **CI,** confidence interval

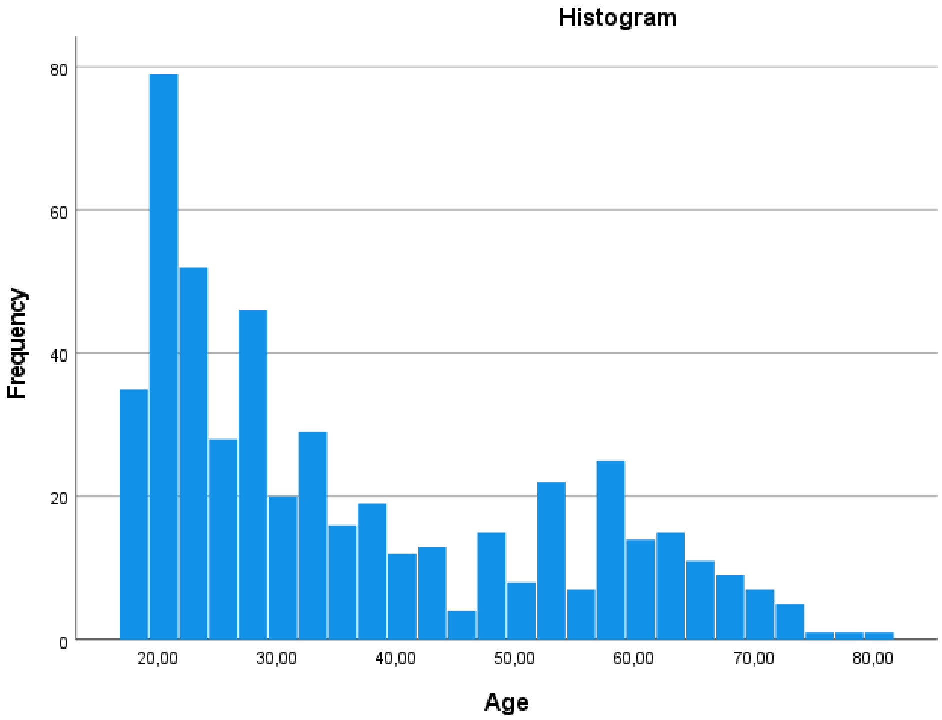

Supplementary Figure 2. Histogram of the distribution of age in the study population.

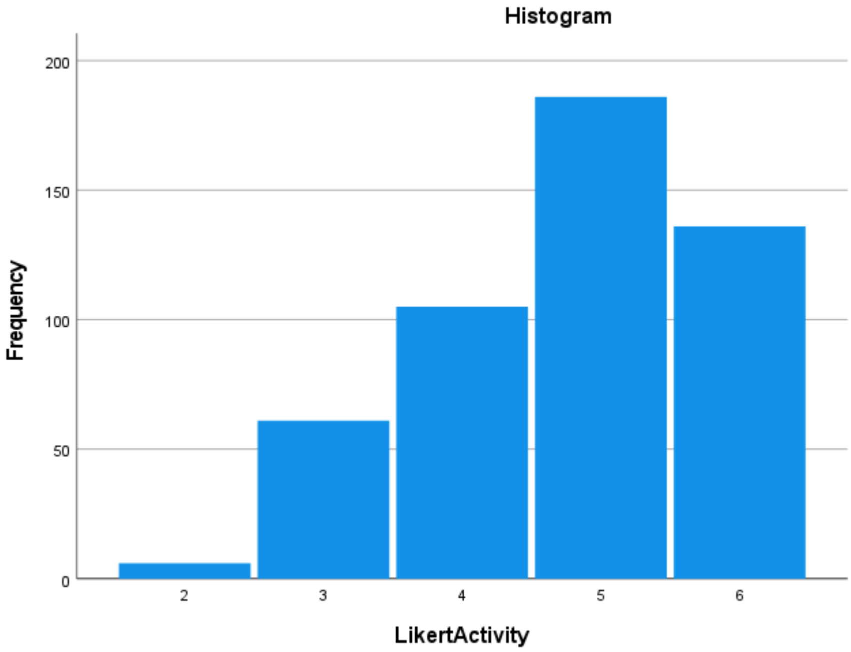

Supplementary Figure 3. Histogram showing the distribution of physical activity level (PAL) in the study population. Likert Scale; 1-6*, 1 = Hardly any physical activity, 2 = Mostly sitting, sometimes walk, easy tasks/play, 3 = Light physical activity for about 2-4 times a week (e.g., fishing, talking, dancing), 4 = Moderate exercise 1-2 hours a week (jogging, swimming, gymnastics), 5 = Moderate exercise at least 3 hours a week (jogging, swimming, gymnastics), 6 = Hard or very hard exercise regularly and several times a week during which the physical exercise is great (jogging, rugby, football).
